# Freshwater Fish Biodiversity Changes Before and After the Indrapuri Dam Construction in the Bihar Region

**DOI:** 10.1093/iob/obag005

**Published:** 2026-03-11

**Authors:** A Khatoon, M K Jaiswal, B Sharma

**Affiliations:** Department of Zoology, Patna University, Patna, Bihar 800005, India; Department of Zoology, C.M.P. Degree College, University of Allahabad, Prayagraj, U.P. 211002, India; Department of Zoology, Patna Science College, Patna University, Patna, Bihar 800005, India

## Abstract

Freshwater biodiversity suffers when dams are constructed. It is important to evaluate how these changes take place, to what level they impact. In this review, we summarized the effects of the Indrapuri Dam’s construction on fish biodiversity over the years on the basis of available studies. We compared the fish biodiversity study done in 1957 before it was built to the studies that were done several years after the dam was ready in 1968. Available information was categorized into five most relevant and other less relevant studies between 1957 and 2023. These studies indicate that fish biodiversity declined drastically after the dam construction. A total of 95 different species of fish lived in the Indrapuri Dam basin before the dam was made; however, only 25 species were noted in 2017 after the construction of the dam. Further, this number increased to 41 in 2023. Several fish species disappeared between 1957 and 2023, notably, those that prefer well-oxygenated and stream-like environments. Biodiversity kept decreasing post-construction for a very long time. The biggest representative order, Cypriniformes, reduced by 75%, followed by the order Siluriformes, which reduced by 61%. Possible reasons for this reduction in diversity could be changes in migratory routes of fishes, the requirement of fast-flowing water, the requirement of upstream movement for spawning, or the requirement of a specific substrate for breeding. Some new fish species showed up in 2014 but were never reported after that. Though many species couldn’t survive these disturbances, fish biodiversity in the dam area has now started improving. By 2023, 12 new fish species belonging to the families Bagridae, Erethistidae, Cichlidae, Cyprinidae, Osphronemidae, and Belonidae had established themselves downstream of the dam. To analyze the reasons for the changing biodiversity in this region, we also reviewed the available studies related to water parameters. Water parameters in the area have improved recently. Some fish species are settling back. If the water parameters continue to improve in line with the recent trend, it is anticipated that the fish biodiversity in the region will improve in the near future.

## Introduction

Construction of dams is one of the most disturbing activities for freshwater fish populations (Kano et al. 2016; Sor et al. 2023). Dams and barrages, while vital for irrigation, flood control, and power generation, are also known to be significant agents of massive changes in riverine ecosystems. The construction of the Indrapuri Barrage on the Son River in Bihar is a prime example of such an intervention. The Son River flows through three states of India, namely Madhya Pradesh, Uttar Pradesh, and Bihar. It is a major right-bank tributary of the Ganga and historically possessed a dynamic as well as diverse ecosystem characterized by a natural flow regime with distinct seasonal variations. The river’s high gradient and ephemeral nature, with roaring flows during the monsoon and disconnected pools in the lean season, used to support a unique assemblage of fish species. With 1407 m (4616 feet) of length, Indrapuri Dam is the fourth-longest dam in the world. The construction of the barrage created a permanent barrier, transforming a free-flowing river into a modified, reservoir-like environment upstream and a fragmented, low-discharge channel downstream. This alteration of the physical habitat, including changes in flow velocity, depth, and substratum, has had a cascading effect on the entire aquatic food web and, most notably, on the composition and abundance of fish species (Joshi et al. 2014; Sor et al. 2023). The dam blocked critical migration routes of fishes that travel upstream for spawning or finding food, which lead to a sharp decline in their population. Stagnant deep-water conditions upstream altered water temperatures leading to lower oxygen levels and affected chemical properties. These new conditions favored generalist or non-native species that have the capacity to tolerate still water. The specialist fish, on the other hand, adapted to well-oxygenated water currents, specific riverbed materials, and natural flood cycles, struggled to survive and reproduce. The dam downstream starved the river of nutrients, and reduced the silt and floodplain inundation (Joshi et al. 2014; Kano et al. 2016). As a result, important habitats were lost. A less robust and diverse aquatic community resulted from the long-term habitat degradation and fragmentation. Isolated fish populations decreased genetic diversity and ultimately led to local extinction of numerous native species (Kano et al. 2016, Sor et al. 2023). This review explores the profound effects of the Indrapuri Barrage on the freshwater fish biodiversity of the Son River and compared the ichthyofaunal diversity before and after its commissioning in 1968. To find out how things have changed over time in this area, we have compared water parameters recently measured in 2023 by us (Khatoon et al. 2026) to the water quality and biodiversity of freshwater fish before and after the construction of the Indrapuri Dam (Motwani et al. 1953; Motwani and David 1957; Rai et al. 2013; Joshi et al. 2014; Demeke and Tassew 2015; Gunasekar and Isaac 2017; Khatoon et al. 2023; Sahu and Tiwari 2023; Sharma et al. 2023a, 2023b, 2023c, 2023d).

## Methodology

### Ethical statement

This is a review, and therefore any ethical permissions were not required.

### Study area description

**Fig. 1:**
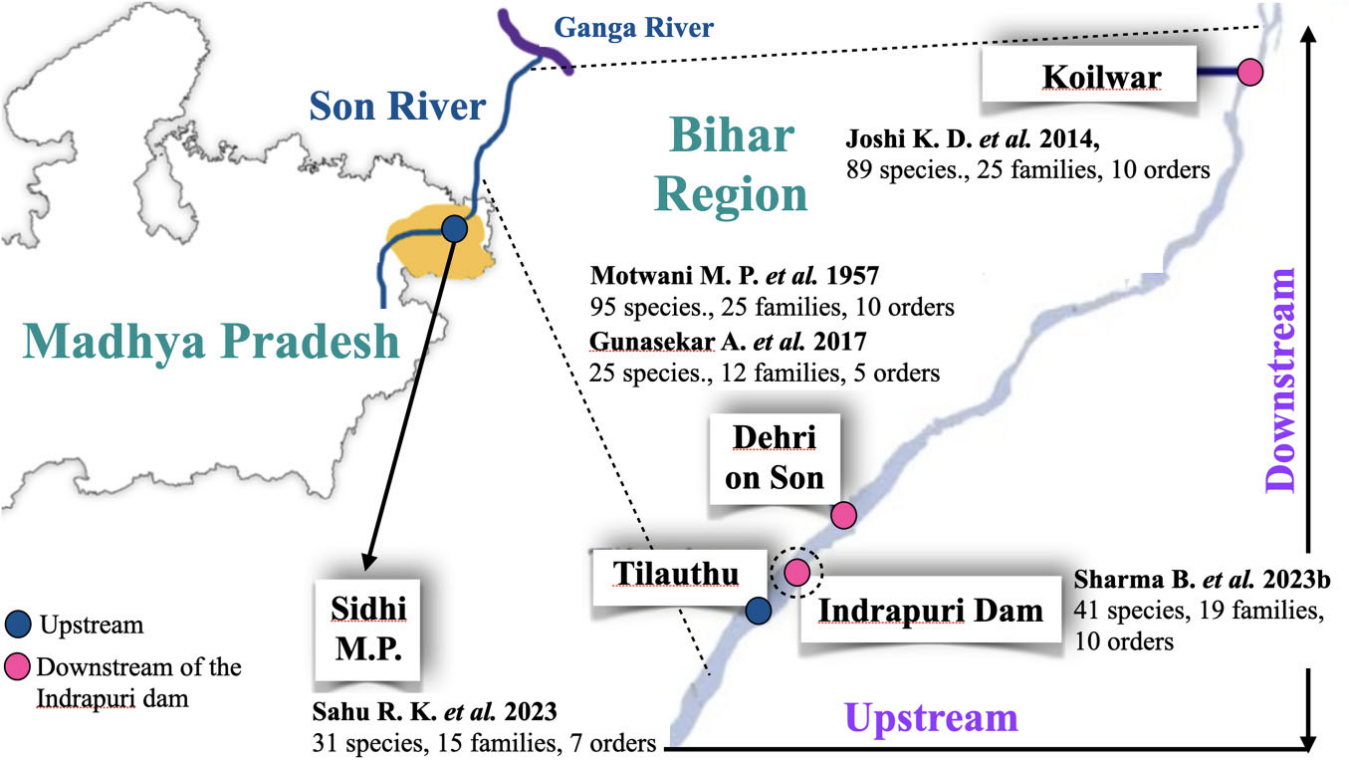
Locations of fish biodiversity studies around the Indrapuri Dam that were included in this review. The sites downstream from the Indrapuri Dam are Dehri on Son and Koilwar. These sites lie in the Bihar region. The upstream study site included in this review lies in the Sidhi district of M.P shown by darker color circle and indicated by solid arrow. The Son River in the Bihar region is magnified within the dotted lines. The light color circles on the Son River represent locations of all the studies done in the Bihar region, downstream of the dam. References of the studies and their finding in brief denoting number of fish species, families and orders are indicated near each study site. One study, Sharma et al. (2023), was done very near the dam both upstream toward Tilathu and downstream before Dehri on Son.

All of the fish biodiversity studies conducted on the Son River upstream and downstream of the Indrapuri Dam are included in this review. Our aim was to comprehend the changes in the Son River’s freshwater fish biodiversity in the Indrapuri Dam region between 1957 and 2023. We chose a brief stretch of the Son River that runs from Tilothu, East Bihar to Koilwar, Bihar. The first study we chose was done in 1957 several years before the dam construction by Motwani and David (1957). The majority of the studies related to fish biodiversity (Joshi et al. 2014, Gunasekar and Isaac 2017, Sahu and Tiwari 2023, Sharma et al. 2023b) were carried out more than 50 years after the 1957 study. The upstream study among these we chose was carried out in the Sidhi district of Madhya Pradesh, which shares its border with Bihar, and was carried out by Sahu and Tiwari (2023). The downstream studies included Joshi et al. (2014), Gunasekar and Isaac (2017), and Sharma et al. (2023b) and were carried out in the Bihar region. A study done in the Madhya Pradesh region that examined 209 km of the Son River (Shukla and Tiwari 2024) was not included in this study, as it lacked details of fish biodiversity and was mostly carried out in Madhya Pradesh. Another study that included data from three rivers (Abhishek 2020) was not included for the same reason. Five major studies that we focused on are listed in Table 1, and their study sites are shown in Fig. 1.

**Table 1:** The list of studies on fish biodiversity included in this review that were carried out on the Son River in the Bihar region and the neighboring Sidhi District in Madhya Pradesh, both upstream and downstream of the Indrapuri Dam, prior to and long after the construction of the dam

Location of study	Year	Species	Family	Order	Reference
Sone River (Bihar)	1957	95	20	10	(Motwani and David 1957)
Sone River (Bihar)	2014	89	25	10	(Joshi et al. 2014)
Sone river (Indrapuri Dam)	2017	25	12	5	(Gunasekar and Isaac 2017)
Sone River (MP)	2023	31	15	7	(Sahu and Tiwari 2023)
Sone River (Indrapuri Dam)	2023	41	19	10	(Sharma et al. 2023b)

### Methods used for specimen collection and species identification

Studies included in this review mostly used cast nets, gillnets, and dragnets for collecting the fish. These are the common methods for catching the fishes in the area. Researchers were mainly dependent on fishermen for specimen collection, and therefore we acknowledge that the fishes that were very small or not so important commercially could have been left out. To identify the species of the fishes, traditional methods as described in the book by K. C. Jayaram and Jhingran, 1977, were used (Khatoon et al. 2023; Sharma et al. 2023b, 2023d). Morphological, morphometric, meristic, and descriptive characters were studied in fresh specimens to avoid misidentification due to shrinkage and color change caused by preservation (Khatoon et al. 2023; Sharma et al. 2023b, 2023d). The fish species reported by the selected study teams were compared in order to ascertain the changes in freshwater fish biodiversity that occurred prior to and following the construction of the Indrapuri Dam. As these investigations had been conducted both upstream and downstream of the dam, it provides a reasonable understanding of the biodiversity changes that occurred post-dam construction.

### Water parameter studies

The water parameters of a body of water determine its biodiversity. Evaluating the relationship between fish biodiversity and water quality metrics is crucial. Nine important studies on water parameters of the Son River in this region were examined. Table 2 provides information on these studies. Only one research, conducted in 1953 by Motwani et al., was conducted prior to the construction of the Indrapuri Dam; the remaining studies were completed years later after the dam construction. A comparison of water conditions that existed in 1953 to how they have changed over time was done. Some studies lack data on some parameters (Table 2), but the data is sufficient to give an idea about how the water quality has changed over time.

**Table 2:** A comparative account of water parameters (temperature, pH, DO, TDS, alkalinity, total hardness, chloride, nitrate, and sulphate) as recorded by different research teams. The first study was done in 1953, and the rest include data collected from 2013 to 2023 from the Son River in the Indrapuri Dam Area. Values shown in the table are the average of the values found round the year.

Location	Temp	pH	DO	TDS	Alkalinity	Hardness	Chloride	Nitrate	Sulphate	References
Dalmianagar Son	27–30	8.5	3.7	–	167	–	–	–	–	(Motwani et al. 1953)
Indrapuri Dam	25.05	8.05	9.5	165.5	213	140.5	55	–	45	(Rai et al. 2013)
Koelwar	–	7.8	–	224.5	–	99.5	344.5	8.5	65	(Maharana et al. 2015)
Dehri on Son	–	7.85	–	265	–	120	480	54	81.5	(Maharana et al. 2015)
Sahar	21.7	7.2	7	407	259	–	21.4	2.72	44.8	(Pandit 2015)
Sahar	29.3	7.1	1.64	–	90.4	179	–	0.21	6.93	(Sharma 2018)
Koelwar	30.1	7.5	3.5	–	68	529	–	0.21	10.9	(Sharma 2018)
Son River Bihar	24	7.3	11.9	–	194	–	1.52	–	–	(Singh 2020)
Indrapuri Dam	23.6	7.72	5.15	115.5	61.35	82.35	26.9	7.69	21.2	(Khatoon et al. 2026, unpublished data)

## Results

As shown in Tables 1 and 2, respectively, this review assesses five important freshwater fish biodiversity studies conducted on the Son River and nine water parameter studies. We examined all the selected fish biodiversity studies up to the species level.

### Comparative study of fish diversity

This review is a comprehensive examination of the fate of freshwater fish biodiversity in the the Son River of the Bihar Region, starting from 1957, approximately 11 years before the dam was erected, to 55 years after the completion of the dam (i.e., up to 2023). Comparing all the available literature, we found that freshwater fish biodiversity was highest in 1957. Later, the Indrapuri Dam construction brought along a severe reduction in freshwater fish biodiversity. All the selected five studies identified 117 freshwater fish species belonging to 30 families and 11 orders in this area (Table 3). We have outlined all of the fishes order-wise for a more systematic and robust examination of biodiversity loss in the area.

**Table 3:** Detailed comparison of the fish orders, families, and species discovered by the five research groups (Motwani and David, 1957; Joshi et al. 2014; Gunasekar and Isaac 2017; Sharma et al. 2023b; Sahu and Tiwari 2023) at various locations on Sone River close to the Indrapuri Dam. Presence of a species is shown by a plus sign “+” whereas absence is shown by a minus sign “−”. Five fishes that established themselves in 2014 and sustained are marked by the darkest color cells and plus sign in the last column of the table S.No. 1, 2, 43, 53, and 64; those that established themselves in 2017 and sustained are marked by the medium color cell and plus sign S.No. 10, 44, 50, and 55. The lightest of all cells with a positive sign in the last column were reported in 2023 only by us S.No. 61, 112, and 115 (Sharma et al. 2023b).

Family	S. No.	Fish species (IUCN Status)	(Motwani and David 1957)	(Joshi et al. 2014)	(Gunasekar and Isaac 2017)	(Sahu and Tiwari 2023)	(Sharma et al. 2023b)
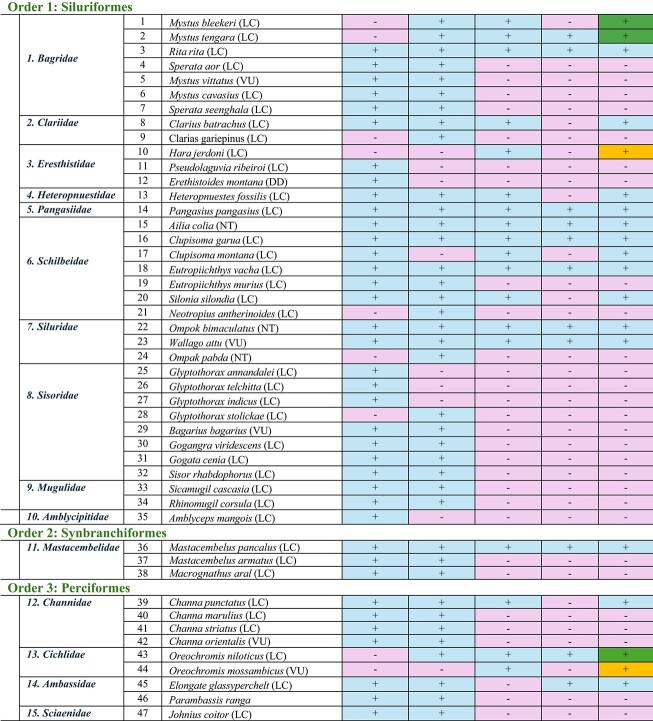							
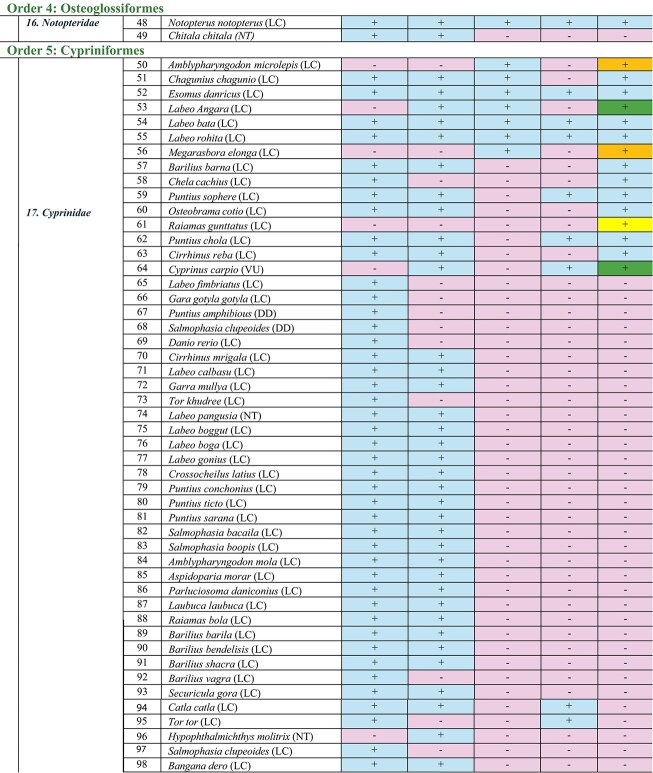							
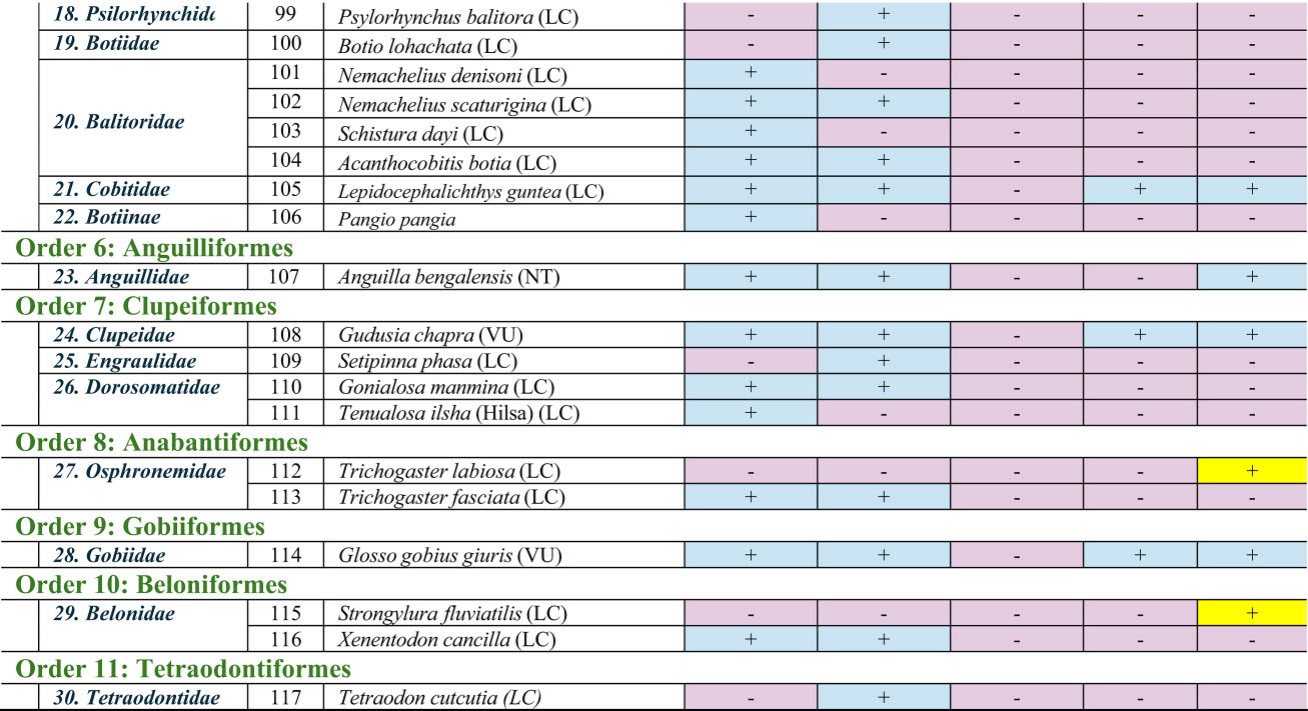							

#### Order Siluriformes

The order Siluriformes is the second most abundant fish order in this area, containing 10 families and 35 fish species. Motwani and David (1957) reported 28 species in 1957, whereas Joshi et al. (2014) reported 27 species. Sharma et al. (2023b) reported only 14 fish species of this order. In 1957, there were five species of this order’s ***Bagridae*** family (Motwani and David 1957). Joshi et al. (2014) reported two more species of this family. These were two newly introduced bagrid catfishes, *Mystus bleekeri* (the striped dwarf catfish) and *Mystus tengara* (the Tengara catfish), that liked post-dam conditions better. The only species of ***Bagridae*** that was present in 1957 and also sustained throughout was *Rita rita*, which prefers slow-moving waters. The family ***Clariidae*** of this order is represented by two species: *Clarius batrachus* and *C. gariepinus. Clarius batrachus* is sustained due to its liking for muddy, slow-moving waters (Sharma et al. 2023c, 2023d). However, a report suggests that *C. gariepinus* is now replacing the native *C. batrachus* species due to its rapid growth, higher fertility, and adaptability (Chand et al. 2021).

In the family ***Erethistidae***, there were two species in 1957. *Pseudolaguvia ribeiroi* likes well-oxygenated cool waters and rocky substrate, and *Erethistoides montana* is a bottom dweller but needs swift currents. Both were probably therefore lost. A third species of this family, *Hara jerdoni*, established itself in dam waters in 2017 and has been observed in the latest study as well (Sharma et al. 2023b). The family ***Heteropneustidae***, with one representative species, *Heteropnuestes fossilis*, and the family ***Pangasiidae***, with one representative species, *Pangasius pangasius*, sustained themselves due to their tolerance for low-quality waters. Of all the ten families of the Siluriformes order, ***Schilbeidae*** was found to be the most resistant. Five out of six members of this family present in 1957 made it to 2023. One species, *Eutropiichthys murius*, was lost after 2014, probably due to overexploitation. Interestingly, *Neotropius antherinoides* was only found in 2014 and never after. *Clupisoma garua* was consistently present before and after dam construction, whereas the presence of *C. montana* wasn’t consistent (Khatoon et al. 2023).

Two families, ***Sisoridae***, containing 8 species, and ***Mugulidae***, containing 2 species, were lost post 2014. In the family ***Sisoridae***, three fishes of *the genus Glyptothorax* were present in 1957 only but never after. They required cool, highly oxygenated waters with strong currents, which was not the case post-dam construction. One species of this genus, *Glyptothorax stolickae*, just appeared in a 2014 study only. Stagnant conditions led to the extinction of this genus in the area. *Bagarius bagarius, Gogangra viridescens, Gogata cenia*, and *Sisor rhabdophorus* faced similar issues and were lost. Out of the two fishes of the ***Mugilidae*** family, *Sicamugil cascasia* is potamodromous, and *Rhinomugil corsula*, or the *Corsula mullet*, is euryhaline. Both of them are rare in India due to overfishing practices and were never found post 2014 due to restricted movements. The family ***Amblycipitidae*** was represented by one species, *Amblyceps mangois*, in this area in 1957; however, it was not reported afterwards. Fishes of this family are commonly known as the torrent catfishes for their liking for fast-flowing, well-oxygenated waters and probably went extinct due to the stagnant water conditions.

#### Order Perciformes

In the order Perciformes, three families (***Scienidae, Ambassidae***, and ***Channidae***) and seven species were present in 1957; another family, ***Chichlidae***, with two members, settled in the new habitat post the Indrapuri Dam construction. However, only four species of three families now represent the order Perciformes (Sharma et al. 2023b). The family ***Scienidae*** had only one member, *Johnius coitor*, but it was not obtained in any study after 2014. The *Johnius coitor*, commonly known as the *coitor croaker*, is an amphidromous fish that used to like shallow waters. The dam created deep waters, and therefore they were lost. The two new species of cichlids, *Oreochromis niloticus* and *O. mossambicus*, were more resistant species therefore sustained themselves. *Oreochromis niloticus* established itself in 2014, whereas *O. mossambicus* established itself in 2017. Both could tolerate stagnant waters with low oxygen and hence found the new ecosystem post-dam construction more suitable. At present four species represent this order, that is, *Elongate glassyperchlet* of the family ***Ambassidae****, Channa punctatus* of the family ***Channidae***, and the two newly established cichlids *O. niloticus* and *O. mossambicus* of the family ***Cichlidae***.

#### Order Cypriniformes

The Cypriniformes order has been consistently the most diverse order of freshwater fishes across all the studies, with 56 species reported overall; Motwani and David (1957) reported 48 species, and Joshi et al. (2014) reported 41 species of this order. However, Gunasekar and Isaac (2017), Sahu and Tiwari (2023), and Sharma et al. (2023b) reported significantly lower numbers of freshwater fish species, that is, 7, 9, and 16 species, respectively. It was found that three-fourths of the species of the Cypriniformes order that were present in 1957 had disappeared by 2023 (Sharma et al. 2023b). The families ***Botiinae, Balitoridae, Botiidae***, and ***Psilorhynchidae*** of this order were lost. Only the family ***Cobitidae***, having one species, *Lepidocephalichthys guntea*, and the family***Cyprinidae*** were sustained in the study area.

The family ***Cyprinidae*** had 43 species in 1957, which reduced to 15 in 2023. *Chela chachius* of ***Cyprinidae***, though present in 1957, reappeared only recently in a 2023 study (Sharma et al. 2023b). The ***Cyprinidae*** family of the Cypriniformes order also shows four new fish species that established themselves post-dam construction that were absent in 1957 before the dam was built. These are *Cyprinus carpio, Barilius barna, Chela cachius*, and *Amblypharyngodon microlepis.* Three new species that appeared in 2014 but could not be observed afterwards are *Botia lohachata* of the ***Botiidae*** family, *Psylorhynchus balitora* of the ***Psilorhynchidae*** family, and *Hypophthalmichthys molitrix* of the ***Cyprinidae*** family. Another new species, *Raiamas guntatus*, established itself very recently in 2023 (Sharma et al. 2023b). In the order Cypriniformes, out of the 52 species reported by all the studies, 36 have disappeared. The torrential rains in 2011 allowed some of them to survive until 2014, but after that, they became unobservable.

Out of the 16 species of this family that survived till 2023, only three species, that is, *Esomus danricus, Labeo bata*, and *Labeo rohita*, were found consistently in all five studies. Four of the five studies reported the presence of *Lepidocephalichthyes guntea, Changunius changunio, Puntius sophere*, and *Puntius chola*. The 1957 study did not report the presence of *Labeo angara, Megarasbora elanga*, or *Cyprinus carpio that appeared in 2014 or after that.* After going missing for a considerable amount of time after 1957*, Chela Chacius* was only observed in 2023. After going missing in 2014 and 2017, *Barilius barna, Osteobrama cotio*, and *Cirrhinus reba* were reobserved again in 2023. *Tor tor* and *Catla catla that were present in 1957* were reported inconsistently in later studies. Following the construction of the dam, *Nemachelius denisoni* and *Schistura dayi* of the ***Balitoridae*** family, as well as *Danio rerio, Labeo fimbraitus, Gara gotyla, Puntius amphibius, Salmophasia clupeoides, Barilius vagra*, and *Tor khudree* of the ***Cyprinidae*** family, were permanently lost and were never seen in the region after the 1957 study.

*Cirrhinus mrigala, Labeo calbasu, Garra mullya, Tor khudree, Labeo pangusia, Labeo boggut, Labeo boga, Labeo goniu, Crossocheilus latius, Crossocheilus latius, Puntius ticto, Puntius sarana, Puntius sarana, Salmophasia boopis, Amblypharyngodon mola, Aspidoparia morar, Parluciosoma daniconius, Laubuca laubuca, Raiamas bola, Barilius barila, Barilius bendelisis, Barilius shacra, Barilius vagra*, and *Securicula gora* of the family ***Cyprinidae*** and order Cypriniformes disappeared post 2014. Heavy rains recorded in 2011 improved biodiversity, as evident by the 2014 study; new fish species were introduced, but later water released from the dam was probably insufficient for them to survive.

#### Minor orders

Fish orders found in the selected studies that consisted of only 1–4 species are mentioned in this review as minor orders. Order **Synbranchiformes**, represented by a single family ***Mastacembelidae***, and three fish species, out of which two, *Mastacembelus armatus* and *Macrognathus aral*, have never been reported after 2014. *Mastacembelus armatus*, also known as the tire track eel, is categorized as endangered due to overexploitation and habitat degradation. It is both an ornamental and edible fish. *Macrognathus aral*, also known as the stripe spiny eel or peacock eel, is categorized as a least concern species but is declining rapidly in India due to overfishing, pressure from exotic species, and habitat destruction. The only surviving species of this order, *M. pancalus*, the barred spiny eel, is categorized as least concern, is indigenous to India, and likes slow, muddy, and benthic waters. These are potamodromous fishes, that is, they need to migrate upstream for spawning. This process was blocked by the dam; however, it has been reported to be present in all five studies.

The order **Osteoglossiformes** is represented by two species of the family ***Notopteridae***. *Chitala chitala* disappeared after 2014, whereas the other, *Notopterus notopterus*, survived and was reported by all five studies. Probably, lack of rocky substrate hindered the spawning process of *the Chitala* genus, leading to their extinction in the area. *Notopterus* could adjust to the changed environment and survived.

The order **Anguilliformes** is represented by only one family, ***Anguillidae***, and only one species, *Anguilla bengalensis*. It was found missing from two studies but has somehow sustained so far. It is a catadromous fish that requires migrating to sea for spawning and therefore is reducing in numbers, as evident by the lack of presence in two studies. It is now classified as near threatened.

The order **Clupeiformes** is represented by three families: ***Clupeidae, Engraulidae***, and ***Dorosomatidae***. Engraulidae had only one species, *Setipinna phasa.* This family was only reported in 2014 but couldn’t be established due to unfavorable conditions and was never reported after that. The family ***Dorosomatidae*** had two representative members in 1957, *Gonialosa manmina* and *Tenualosa ilisha* (Hilsa). Although *Gonialosa manmina* could only make it until 2014, *Tenualosa ilisha* (Hilsa) was never reported after 1957. Since Hilsa is very important commercially, it was a major loss, so much so that old local fishermen mentioned that they missed its presence. Hilsa is an anadromous fish that comes to freshwaters for spawning, and with the altered routes post-dam construction, the migration was blocked, and this fish was never seen afterwards. Only one family of this order, ***Clupeidae***, with one representative species, *Gudusia chapra*, could make it to 2023, though it was also missing in the 2017 study.

In the order **Anabantiformes**, represented by the single family ***Osphronemidae***, *Trichogaster fasciata* was shown to be present only till 2014; however, in 2023, another new species of this family, *Trichogaster labiosa* was reported which likes sluggish, shady waters and is sensitive to water quality, especially nitrate levels.

The order **Gobiiformes** is represented by only one family, ***Gobiidae***, and only one species, *Glossogobius giuris*. It is classified as vulnerable and prefers stagnant waters. It was reported by all studies except the 2017 study.

The order **Beloniformes**, had only one family ***Belonidae***, and only one species, *Xenentodon cancilla*, in 1957 and 2014. It was never reported after 2014; however, another new species of this family, *Strongylura fluviatilis*, was recently reported in a 2023 study.

The new order **Tetraodontiformes**, was reported only in 2014 by a single species, *Tetraodon cutcutia*, of the family ***Tetraodontidae***. It was never reported afterward. This fish is an ornamental fish and is overharvested for that reason.

**Fig. 2:**
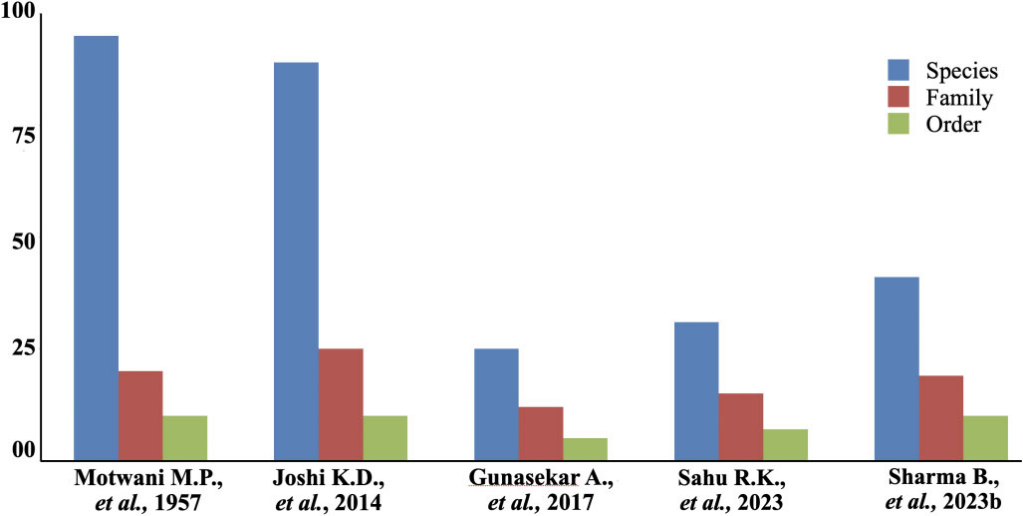
Changes in fish biodiversity that have occurred in the Son River as reported by various studies. Of the three columns on the *x*-axis, the first one indicates the number of fish species, the second to the right the number of fish families, and the third to the right the number of fish orders documented in that study.

The graph shown in Fig. 2 represents the overall changes that took place in the number of freshwater fish species, their families, and orders. There is a sharp decline in the number of species until 2017, and then the number of species increases slowly. Orders and families show similar trends.

### Comparative water parameters

In the context of freshwater ecosystems, water quality parameters serve as the fundamental determinants of habitat suitability and species distribution, directly influencing the assembly and maintenance of fish biodiversity. Physico-chemical variables act as invisible physiological boundaries that dictate the survival, metabolic rates, and reproductive success of aquatic fauna. Fish are poikilothermic and highly sensitive to osmotic fluctuations. Even marginal deviations in water parameters can lead to physiological stress, altered competitive dynamics, or localized extinctions of stenotropic species. Fish biodiversity in the area we studied has fluctuated drastically over the years. We therefore searched for the available literature to understand if the studies related to water parameters provide some insights into the fish biodiversity fluctuations we observed. Nine studies related to water parameters were reviewed. The latest data was collected by Khatoon et al. (2026). A summary of the selected nine studies on water parameters is given in Table 2.

#### Water temperature

**Fig. 3:**
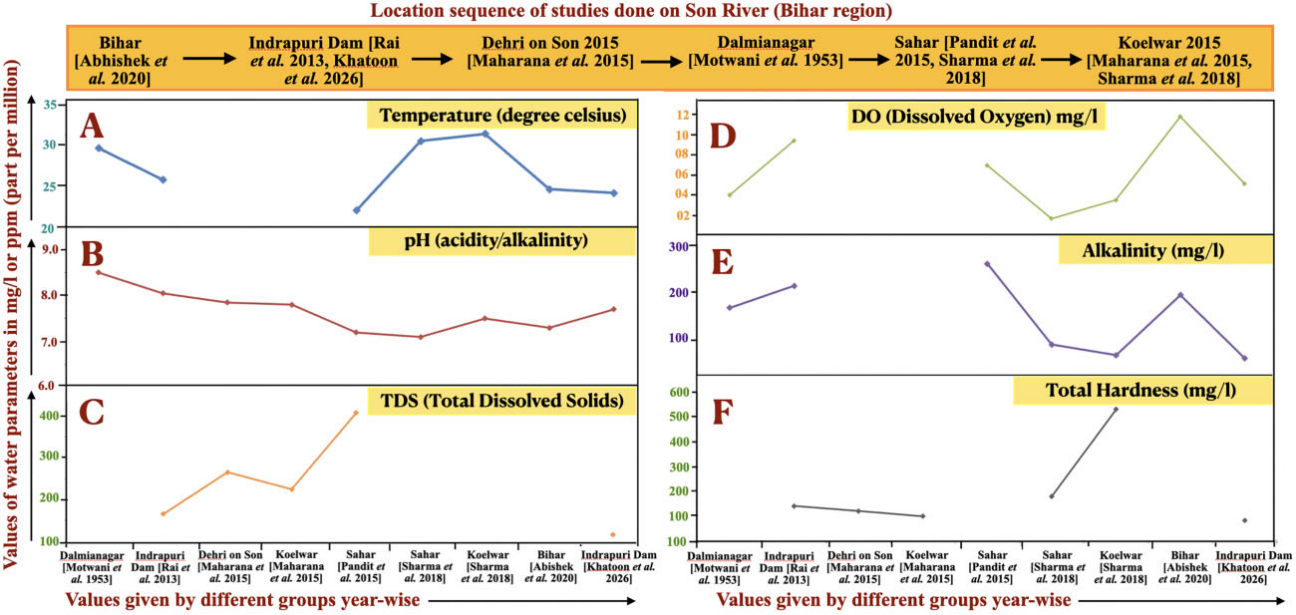
Summary of all the water parameter studies done in the stretch of the Son River that lies in Bihar before it merges with the River Ganga. Locations of the studies we selected are in the sequence shown in the top panel. Panel A, temperature (in degrees Celsius) data recorded in various regions by different groups; (B) pH levels in those locations; (C) total dissolved solids (TDS); (D) dissolved oxygen (DO) levels; (E) alkalinity values; and (F) total hardness values. Units of measurement are given on the *Y* axis in ppm (part per million) or mg/L, whereas the *X* axis represents the location of the study, the year, and the reference numbers of the research articles from which the data was taken. Studies are arranged in an ascending sequence of years.

Temperature influences the physiological and biochemical behavior of the fishes. Increasing temperature speeds up metabolism and reduces oxygen solubility. Fish keep their body temperatures between 0.5 and 1°C above or below the water temperature. Most fish species have optimal immunological function at 15°C (Stone and Thomforde 2004). Water temperature at Indrapuri Dam ranges between 17 and 30.2°C (Khatoon et al. 2026), like other previous findings as shown in panel A of Fig. 3.

#### pH

In 1957, the pH range recorded in the area was 6.9–9.2, with the lowest values at Hurka and the highest at two nearby locations, Makrain and Darihat (Motwani et al. 1953). Water of the Indrapuri Dam area was never found to be acidic (Fig. 3, panel F). October had the lowest pH (7.2), while April and May had the highest (8.6) pH values (Khatoon et al. 2026). pH between 6.5 and 8.5 is thought to be optimal for fishes (Santhosh and Singh 2007; Kiran 2010). In 2023, pH seems to have lowered toward a more neutral state. Nonetheless, it is always alkaline (Figure, panel B).

#### Total dissolved solids

TDS toxicity is stage-specific, such that it affects fertilization in salmonids (Abowei 2010). In 1957, the value of suspended solids was 1218 ppm in the Makrain area and 820 ppm in the Hurka area near Indrapuri Dam, due to industrial effluents. In 2023, TDS levels varied from 143 to 188 mg/L (Khatoon et al. 2026), and no other studies report values more than 400 mg/L in the area from 2013 to 2023 (Fig. 3, panel C). Fish are thought to be healthy in water with TDS levels under 400, indicating that the ecosystem is improving. This could be one of the reasons for the recent slight increase in the fish biodiversity.

#### Dissolved oxygen

Cyprinids prefer 6–8 mg/L of DO and exhibit suffocation symptoms between 1.5 and 2.0 mg/L (Santhosh and Singh 2007). Although 4 mg/L is sufficient to keep catfishes alive (Boyd and Lichtkoppler 1979), they die at 0.3 mg/L. Water bodies must have 5.0 mg/L of DO to sustain fish (Kiran 2010). In 1957, due to industry influence, the Makrain area of the Indrapuri Dam had the lowest DO of 0.2 mg/L, which was lethal for fishes. The rest of the area was comparatively pollution-free and supported fish health. For all studies in the area, it remained between 2 and 12 mg/L (Fig. 3, panel D). Summer months showed the lowest DO levels, whereas winter months had the highest (Rai et al. 2013). The most recent values of DO record in the area ranged between 4 and 6.4 mg/L (Khatoon et al. 2026). DO levels in ppm (parts per million) and their impact on fish output are shown in Table 4. Data suggests that the DO levels around the Indrapuri Dam now are best suited for freshwater fishes. This is also evident by the slight increase of biodiversity in 2023 compared to 2017.

**Table 4:** The DO levels in ppm (parts per million or mg/L) and their effects on fish health. Values between 5 and 14 ppm are preferred by fishes. The DO levels in the Indrapuri Dam region are almost at the ideal levels in the 2023 study.

S. No.	DO level (ppm or mg/L)	Impact on fish health and production and references
1	>14 or between 1 and 3	Harmful. (Santhosh and Singh 2007)
2	<5	Excellent. (Santhosh and Singh, 2007)
3	0.2 (Makrain)–7.2	The Makrain area had the least DO due to the effluents dumped by Rohtas industries till 1984. (Motwani et al. 1953)
4	4.0–6.4	(Khatoon et al. 2026)
5	2.0–12	Values from 2013 to 2023

#### Alkalinity

Alkalinity of a water body increases by leaching, photosynthesis, denitrification, sulphide reduction, respiration, nitrification, sulphide oxidation, and, to a lesser extent, by evaporation and decomposition (Beitinger and Fitzpatrick 1979; Ekubo and Abowei 2011; Singh 2020). Summer months have the highest alkalinity, and winter months the lowest. Alkalinity values ranged from 60 to 260 mg/L (Fig. 3, panel E, and Table 5). In 2023, it varied between 4 L and 82 mg/L, indicating a declining trend and the best conditions for fishes in the past decade.

**Table 5:** The alkalinity variations and their impact on fish production. The values are expressed in parts per million, or ppm. Different fish species require different ideal values for optimal production. Alkalinity in the range of 75–200 ppm is considered best for fish health, and for the past few years it has remained less than 200 ppm.

S. No.	Total Alkalinity (in ppm)	Impact on fish health and production	References
1	0–20	Low production, poor condition.	Swann (1997) and Moyle (1949)
2	20–150	Suitable for fish production.	(Boyd and Lichtkoppler 1979)
3	75–200	Ideal for aquaculture.	(Wurts and Durborow 1992; Bhatnagar et al. 2004)
4	50–300	Desired range of CaCO3.	(Santhosh and Singh 2007)
5	>300	Unfavorable due to lack of CO2	Stone et al. (2004) and Bhatnagar et al. (2004)
6	216	Alkalinity of Makrain area was highest (216) for rest (118–160)	(Motwani et al. 1953)
7	41–81	Alkalinity found in suitable range.	(Khatoon et al. 2026)
8	< 200	Alkalinity in suitable range.	Last few years: 2018–2023 (Sharma 2018; Singh 2020)

#### Total hardness

A minimum of 20 ppm of hardness is necessary, and a range of 30–180 mg/L is ideal (Swann 1997; Stone et al. 2004; Abhishek 2020) for fishes. The recommended hardness values needed for fish health are between 75 and 150 ppm or mg/L, although there are some variations depending on the kind of fish. Less than 20 ppm causes stress (Bhatnagar et al. 2004), while more than 300 ppm is lethal. Total hardness values ranged from 123 to 500 mg/L in previous studies (Fig. 3, panel E, and Table 6), and in 2023 it was in the range of 62–102 mg/L (Khatoon et al. 2026), indicating a significant drop in hardness. Hardness of water around Indrapuri Dam is optimum for fishes in 2023.

**Table 6:** Impact of changes in water hardness on fish health and production. Values are reported in ppm (parts per million). The values of Indrapuri Dam are within the recommended range. The impact of changes in water hardness on fish health and production is given in Table 6. In 2013 the alkalinity values recorded were close to the maximum values within the optimum range. However, in 2023 they decreased and approached the lower values of the optimum range.

S. No.	Total hardness in ppm	Impact on fish health and production	References
1	<20 or >300	Not favorable for fish. Causes stress	(Swann 1997; Bhatnagar et al. 2004)
2	30–180 75–150	Favorable for fish culture Ideal for fish.	(Boyd and Lichtkoppler 1979; Swann 1997; Santhosh and Singh 2007)
3	50–150	Optimum range of CaCO3	(Stone et al. 2004)
4	123–158	Indrapuri Dam area 2013.	(Rai et al. 2013)
5	62–102	Indrapuri Dam area 2023	(Khatoon et al. 2026)

#### Turbidity

Turbidity levels between 30 and 40 cm are considered optimal for fish culture (Santhosh and Singh 2007). The unit of measurement for turbidity is the Nephelometric Turbidity Unit (NTU). Fish in freshwater generally tolerate turbidity levels up to 50 NTU. In 1953, due to the waste from the paper pulp factory, the turbidity of the Makrain area was 300 NTU, whereas Dehri on Son was only 10 NTU (Motwani et al. 1953). Post factory shutdown, water condition improved considerably to 2.7–4.2 NTU (Khatoon et al. 2026), indicating sustainable circumstances for fishes. We could only find two studies (Table 7) to compare turbidity.

**Table 7:** Values of turbidity, conductivity, and nitrate levels observed in different studies and their impact on freshwater fishes. Current values of all three indicate that the water is favorable for freshwater fish development.

S. No.	Water Parameters	Levels and metrics	Impact on fish health and production	References
1	Turbidity	30–80 cm	Good for fish health.	(Bhatnagar and Devi 2013)
2		30–40 cm	Optimum productivity.	(Santhosh and Singh 2007)
3		2.7–4.2 NTU	Favorable for fish.	(Khatoon et al. 2026)
4		10 NTU (Dehri and rest) -300 NTU (Makrain)	Due to paper pulp factory. Makrain area had maximum (300) whereas other (10–60) which changed later	(Motwani et al. 1953)
5	Conductivity	30–5000 µ S/cm	Acceptable for fish culture.	(Stone et al. 2004)
6		148–251 µS/cm	Favorable for fish.	(Khatoon et al. 2026)
7		270 (Dehri)–520 µS/cm (Makrain, Hurka)	Conductivity was high in Makrain and Hurka area.	(Motwani et al. 1953)
8	Nitrate	90 mg/L	Nontoxic to fish. No health hazards.	(Stone et al. 2004)
9		0.1–4.0 mg/L	Favorable for fish	(Santhosh and Singh 2007)
10		1.68–13.72 mg/L	Favorable for fish.	(Khatoon et al. 2026)

#### Conductivity

Conductivity is measured in units of micro siemens per centimeter (µS/cm). Fishes can tolerate conductivity levels between 30 and 5000 µS/cm (Stone et al. 2004). Values for conductivity ranged from 148 to 251 µS/cm in 2023 (Khatoon et al. 2026), suggesting it was in a healthy range for the fishes (Table 7). Other studies were lacking data on this one as well.

#### Nitrates

Value of nitrates were also found to be in range in 2023 to support healthy growth of fishes in the study area (Khatoon et al. 2026). No other groups report nitrate values (Table 7).

After examining the water characteristics of Indrapuri Dam, we see that there were significant variations from 1953 to 2023 in water parameters (Keller et al. 2019). pH values have moved toward a more neutral state recently (Rai et al. 2013; Khatoon et al. 2026). DO markedly dropped from 9.5 mg/L in 2020 to 5.15 mg/L in 2023. There is a decline in TDS, alkalinity, and hardness, suggesting better water quality as the values are approaching optimum. Over the last few years, there has been an overall improvement in the quality of the water, which apparently led to a little rise in biodiversity of freshwater fishes in 2023 (Sharma et al. 2023b). Table 2 and Fig. 3 provide a comparison of the changes in water parameter values over time. The disjointed and inconsistent data availability obstructs comprehensive analysis; however, it provides sufficient evidence to suggest that water parameters have improved over time. This highlights the necessity for more systematic and continuous monitoring efforts in the future to enhance aquatic environments for fish culture, thereby supporting our finding of increased fish biodiversity in the region.

## Discussion

The available research on fish biodiversity and water characteristics in the Son River before and after the Indrapuri Dam was built in the Bihar region is compiled in this review. A total of 95 species of fish were found by researchers prior to the construction of the dam in 1957. Out of which, 41 fish species were identified in 2023 (Sharma et al. 2023b). Recent investigations have shown a discernible trend of decreased species counts (Gunasekar and Isaac 2017; Sahu and Tiwari 2023; Sharma et al. 2023b). Following the construction of the Indrapruri Dam, a sharp decrease in fish biodiversity was noted. Reduced water levels and sediment/nutrient loss downstream of the dam, stagnant deep waters and decreasing oxygen levels upstream of the dam, obstruction of migratory fish passage upstream for spawning, and other alterations in habitat conditions are some potential causes (Joshi et al. 2014). Pollution from the Rohtas factories in Dalmianagar was another factor that led to this loss. Paper, cement, chemical, vanaspati, and sugar factories were among them. The Makrain and Hurka regions of Dalmianagar were the most polluted until these factories closed in 1984 because they all dumped their toxic effluents into the Son River (Motwani et al. 1953). Although there is no data on fish diversity in this region from 1957 to 2014, it is possible that fish biodiversity may have drastically decreased downstream shortly after the dam was built due to the contaminated and limited waters, and that diversity restoration may have begun after 1984 (Rai et al. 2013; Pandit 2015; Singh 2020; Khatoon et al. 2026). The intense water rise brought on by the heavy rains in 2011 (Joshi et al. 2014) further enhanced the environment, and new freshwater fish species were found in 2014 as a result of fewer contaminated waters and the gradual ecological restoration taking place in this region. With 56 species out of 117, the Cypriniformes order was consistently the most diverse across all investigations, followed by the order Siluriformes. In the past six to seven decades, 61% of the species of the order Siluriformes and 75% of the order Cypriniformes have vanished. Fish that migrate, such as *Hilsa, Bagarius*, and *Pangasius*, showed a dramatic decline in population. Fishes that liked stagnant waters started emerging, whereas those liking well-oxygenated and running water disappeared. Twelve new species settled down in the dam waters that were not present before the dam construction. Among these fishes were two bagrid catfishes (*Mystus bleekeri* and *Mystus tengara*), two cichlids (*O. niloticus* and *O. mossambicus*), and two cyprinids (*Labeo angara* and *Cyprinus carpio*). All of these emerged in 2014 except *O. mossambicus*. It, along with two cyprinids (*Amblypharyngodon microlepis* and *Megarasbora elanga*) and *Clarius gariepinus*, emerged in 2017. *Trichogaster* labiosa, the cyprinid *Raiamas guntatus*, and *Strongylura fluviatilis* were reported in 2023 (Sharma et al. 2023b). All these liked the ecological conditions better post-dam construction. Apparently, a shift toward more resilient species is taking place. The emergence of omnivorous *Clarias gariepinus* is noteworthy. These fishes are voracious eaters and not of much economic value, but they are replacing the existing native *Clarias batrachus* species very fast (Chand et al. 2021), which is alarming.

## Conclusion

The construction of the Indrapuri Barrage on the Son River in Bihar has had a profound and detrimental impact on its aquatic ecosystem. The barrage altered the river’s natural flow, fragmented habitats, and, most critically, blocked ancient migratory routes for many fish species. Several native fish species have either completely disappeared from the waters, or their populations have declined so drastically that they are now considered locally extinct or critically endangered. The freshwater fish biodiversity declined for almost 50 years post-dam construction. There’s a noticeable trend of lower species counts in the more recent studies (Gunasekar and Isaac 2017; Sahu and Tiwari 2023; Sharma et al. 2023b) compared to before dam construction (Motwani and David 1957), especially prominent in the orders Cypriniformes (75%) and Siluriformes (61%). Data suggests that most fish species experienced significant pressures and decline in the sampled region. It also aligns with the broader trend of freshwater fish species decline worldwide due to pollution and other anthropogenic activities (He et al. 2019). Overall, after more than 50 years of decline, the biodiversity of freshwater fishes in the region has now started to improve. Twelve new species that like the new ecosystem post-dam construction have emerged. Five species in 2014, 4 in 2017, and 3 in 2023 (Joshi et al. 2014; Gunasekar and Isaac 2017; Sharma et al. 2023b). If current ecological circumstances continue to improve, fish biodiversity is predicted to continue increasing. The construction of a fish ladder, which is absent from the Indrapuri Dam, could have lessened the loss of migratory species. To improve the water body, new fish species should be manually added. This is also necessary for the socio-economic upliftment of the fishermen that live in this area.

## Data Availability

Any data asked for will be provided on request.
